# Web-Based Surveillance of Public Information Needs for Informing Preconception Interventions

**DOI:** 10.1371/journal.pone.0122551

**Published:** 2015-04-16

**Authors:** Angelo D’Ambrosio, Eleonora Agricola, Luisa Russo, Francesco Gesualdo, Elisabetta Pandolfi, Renata Bortolus, Carlo Castellani, Faustina Lalatta, Pierpaolo Mastroiacovo, Alberto Eugenio Tozzi

**Affiliations:** 1 Multifactorial Disease and Complex Phenotype Research Area, Bambino Gesù Children’s Hospital IRCCS, Rome, Italy; 2 Office for Research Promotion, Department of the Hospital Management and Pharmacy, Verona University Hospital, Verona, Italy; 3 Cystic Fibrosis Centre, Verona University Hospital, Verona, Italy; 4 Clinical Genetics Unit, Fondazione IRCCS Cà Granda Ospedale Maggiore Policlinico, Milan, Italy; 5 Alessandra Lisi International Centre on Birth Defects and Prematurity, Rome, Italy; NIDCR/NIH, UNITED STATES

## Abstract

**Background:**

The risk of adverse pregnancy outcomes can be minimized through the adoption of healthy lifestyles before pregnancy by women of childbearing age. Initiatives for promotion of preconception health may be difficult to implement. Internet can be used to build tailored health interventions through identification of the public's information needs. To this aim, we developed a semi-automatic web-based system for monitoring Google searches, web pages and activity on social networks, regarding preconception health.

**Methods:**

Based on the American College of Obstetricians and Gynecologists guidelines and on the actual search behaviors of Italian Internet users, we defined a set of keywords targeting preconception care topics. Using these keywords, we analyzed the usage of Google search engine and identified web pages containing preconception care recommendations. We also monitored how the selected web pages were shared on social networks. We analyzed discrepancies between searched and published information and the sharing pattern of the topics.

**Results:**

We identified 1,807 Google search queries which generated a total of 1,995,030 searches during the study period. Less than 10% of the reviewed pages contained preconception care information and in 42.8% information was consistent with ACOG guidelines. Facebook was the most used social network for sharing. Nutrition, Chronic Diseases and Infectious Diseases were the most published and searched topics. Regarding Genetic Risk and Folic Acid, a high search volume was not associated to a high web page production, while Medication pages were more frequently published than searched. Vaccinations elicited high sharing although web page production was low; this effect was quite variable in time.

**Conclusion:**

Our study represent a resource to prioritize communication on specific topics on the web, to address misconceptions, and to tailor interventions to specific populations.

## Introduction

As recently emphasized in the WHO Global Action Plan for the Prevention and Control of Non-communicable Diseases 2013–2020 [1], risk identification, adoption of preventive measures and promotion of healthy behaviors during the preconception period may prevent the emergence of adverse pregnancy outcomes (APOs) [2,3].

In 2008, the American College of Obstetricians and Gynecologists (ACOG) issued a set of guidelines for preconception interventions [4,5]. The core of the recommendation set included folic acid supplementation, adoption of healthy lifestyles and medical care. These recommendations are also valid during pregnancy: defining a set of behavioral and medical interventions before pregnancy minimizes the risk of APOs during the whole reproductive period and maximizes the effect of preventive interventions adopted during pregnancy [4,6].

In Italy, every year approximately 90,000 newborns are born either preterm or with birth defects, with a rate of nearly 250 per day [7]. Recently, the Italian Ministry of Health supported a project for preconception health promotion, for which the ACOG guidelines were used as a reference and innovative information strategies were explored (www.pensiamociprima.net).

International initiatives for promotion of preconception health rarely focused on the whole set of ACOG recommendations [8] and often specifically promoted folic acid supplementation [9,10]. In most cases, information needs of target populations were assessed through interviews, surveys, and focus groups [11,12], yielding results that might not be generalizable to all population segments. Moreover, the public’s information needs may vary over time.

On the other hand, a number of health interventions have been conducted on the Internet, exploiting modern online marketing techniques [13–16]. Internet-based interventions encompass identification of the population at risk and its dynamic needs and interests [17,18], and implementation of actions based on these data [15,19], in order to tailor interventions to the appropriate targets [20].

To our knowledge, the Internet has never been used to identify the population’s information needs regarding preconception health.

To this aim, we developed a semi-automatic, web-based monitoring system to inform public health interventions and applied it to preconception health in the Italian population.

## Material and Methods

### Study design

We developed a semi-automatic, web-based system for monitoring preconception care contents searched, published on web pages and shared on social networks by Italian Internet users.

To this aim, we revised the guidelines for preconception care issued by the ACOG [5] and we selected the following topics: Alcohol Consumption, Chronic Diseases, Environmental Exposures, Folic Acid Supplementation, Genetic Risk, Illicit Drugs, Infectious Diseases, Medications, Nutrition, Physical Activity, Sexually Transmitted Diseases (STDs), Smoking, Vaccinations. For each topic, we selected a set of keyword combinations which were used to monitor the web (see “Keyword selection and probe set up”). We focused on Italian language contents generated from September 2013 to April 2014. Data on search volumes, number of published web pages and sharing volumes of such web pages on social networks were recorded and described for each topic. We also described the correlation between information search, publication, and sharing volumes. S1 Algorithm shows a chart with a summary of the three components of the study.

### Keyword selection and probe set up

Firstly, we selected a set of four basic, preconception-related keywords (namely: pregnancy, preconception, conception, prenatal). Secondarily, we selected a second set of keywords which were specific for each topic of the ACOG recommendations. For a complete list of the keywords, see S1 Supplement.

Thirdly, for each of the preconception topics, we combined the two sets of keywords into combinations hereafter referred to as “probes”, which were used to identify relevant preconception-related searches and contents.

Probes were adjusted and expanded through the Google Keyword Planner Tool (www.adwords.google.com), an online application which suggests keyword combinations on the basis of the actual usage of the search engine by Google users.

### Google search monitoring

To investigate search volumes, we used metrics measured through Google Adwords (www.adwords.google.com). We submitted the probes for each topic to the Google Adwords’ Keyword Planner Tool, from which we obtained the related search queries which had actually been used by Italian Internet users. Through the same tool we obtained monthly search volumes for each query. We manually reviewed the results to filter out the search queries which were not related to the topics derived from the ACOG recommendations, and to identify sub-topics.

### Web page content monitoring

Through our predefined probes, we set up a retrieval system for identifying web pages reporting preconception care contents through the Trendiction S.A. Talkwalker Alerts tool (www.talkwalker.com/alerts/). Talkwalker Alerts is a query-based alerting system which crawls the Internet for recently published web pages containing text that matches specific queries and provides results in a Really Simple Syndication (RSS) stream accessible via URL. Resulting web pages were automatically stored in a dedicated web platform that was built using Wordpress (wordpress.org). The Pods plugin (pods.io) was used for data management.

For each web page, we collected the Alexa Score (www.alexa.com), which ranks a web site on the basis of the number of visitors and page views: the lower the rank, the higher the popularity of the web site. We selected the web pages from web sites which had a rank lower than the 25th percentile as retrieved in September 2013.

The database automatically excluded web pages which a) were unreachable; b) did not contain the keywords in the main content of the page; c) were duplicates.

The selected web pages were automatically classified into at least one of the preconception topics according to the keywords found in the text.

Moreover, the selected web pages were manually reviewed by three trained Authors (ADA; EA; LR) who classified them as relevant when explicitly containing preconception care contents. Relevant resources were then classified by page type into: a) discussion pages (pages from web forums, Q&A sites and similar, whose content was written by users communicating with other users); b) information pages (pages which contained unidirectional information, not meant for interaction). Finally, information pages were evaluated for consistency with the ACOG recommendations and classified as: a) correct (i.e. reporting information which was complete and fully consistent with the ACOG recommendations); b) incorrect pages (i.e. reporting incorrect, partially correct, or incomplete information according to the ACOG recommendations).

### Monitoring of information shared on social networks

On the 28th of July 2014, 90 days after closing the web page monitoring activity, we processed the URLs of the retrieved information pages through the sharedcount.com service (www.sharedcount.com), a tool providing the volume of social interactions on the following social networks: Facebook, Twitter, Google+, LinkedIn, Stumble Upon, Reddit, Delicious, Buzz, Pinterest, Diggs.

### Data analysis

Regarding Google searches, we calculated the number of queries related to each topic, and their proportion over the total number of queries for all topics. We also calculated the total search volume generated by all the queries related to each topic and its proportion over the search volume generated by all queries for all topics.

Regarding web pages, for each topic we calculated the volume of web pages containing preconception care contents, and the proportion of such pages over the total volume of pages for all topics. We also described the frequency of discussion pages and information pages for each topic. For information pages, we calculated the proportion of web pages that reported correct recommendations by topic.

Regarding sharing on social networks, for each topic we calculated the total volume of sharings on social networks. For each topic, we also computed a sharings/page ratio as the ratio between the total number of social interactions and the total number of information pages. This ratio represents an indicator of virality. We also reported the volume and proportion of sharings by social network.

We built a first scatterplot to analyze the relationship between searched and published information on each topic. A second scatterplot was built to analyze the sharing pattern for each topic through page sharing. To this end, we normalized the monthly volumes for each topic transforming them into z-scores. We used standard deviations of monthly data for each topic using ellipses to represent variability over time. A regression line was plotted to indicate the relative trend between indicators.

## Results

### Google searches

A total number of 29,132 queries related to our probes were generated. After manual evaluation, 27,325 (93.8%) were excluded since not related to our topics. The 1,807 included queries generated a total of 1,995,030 searches during the study period. Table 1 shows Google search data by topic and their corresponding volumes.

**Table 1:** Google search data by topic and their corresponding volumes.

**Topic**	**Search queries** ^a^	**Search volumes** ^b^	**Explicit Preconception search volumes** ^c^
**Nutrition**	549 (30.4%)	444640 (22.3%)	13120 (2.95%)
**Folic Acid**	180 (9.96%)	425230 (21.3%)	12890 (3.03%)
**Genetic Risk**	183 (10.1%)	330430 (16.6%)	4910 (1.49%)
**Infectious Diseases**	231 (12.8%)	257280 (12.9%)	2100 (0.816%)
**Chronic Diseases**	225 (12.5%)	241800 (12.1%)	3680 (1.52%)
**Medications**	107 (5.92%)	82740 (4.15%)	1420 (1.72%)
**Physical Activity**	76 (4.21%)	77880 (3.9%)	680 (0.873%)
**Smoking**	53 (2.93%)	51660 (2.59%)	690 (1.34%)
**Alcohol**	46 (2.55%)	29340 (1.47%)	0 (0%)
**Environmental Exposure**	74 (4.1%)	29130 (1.46%)	0 (0%)
**Sexually Transmitted Diseases**	32 (1.77%)	10800 (0.541%)	1230 (11.4%)
**Vaccinations**	26 (1.44%)	7720 (0.387%)	770 (9.97%)
**Illicit Drugs**	25 (1.38%)	6380 (0.32%)	0 (0%)
**Total**	1807 (100%)	1995030 (100%)	41490 (2.08%)

^a^ Percentage on total number of queries.

^b^ Percentage on total search volume.

^c^ Percentage on topic volume

Information on Nutrition is the most searched, followed by Folic Acid and Genetic Risk. The topics with the lowest search volumes were STDs, Vaccinations, and Illicit Drugs.

We identified by manual review a total of 255 sub-topics (S2 Supplement). Among the sub-topics with the highest search volumes were: weight control, diabetes, hypertension, toxoplasmosis, cytomegalovirus, Down syndrome, paracetamol, antibiotics, smoking, alcohol consumption, use of cosmetics and rubella immunization.

Of note, most searches regarded both preconception and pregnancy. Only 41,490 (~2%) searches were uniquely and unequivocally associated with the preconception period only. Most of the searches focusing on preconception issues regarded fertility (58%).

### Web pages

The monitoring system for preconception contents published on the web captured 5,717 web pages, which were manually evaluated. Although matching the study queries, 5,290 (92.6%) did not contain any information on preconception care related to the ACOG recommendation (eg.: news, gossip, movie plots, recipes). The remaining 420 (7.4%) were then analyzed for type of web page (discussion/information page) and consistency with ACOG recommendations. As for page type, we found that 151 (35.9%) were discussion pages and 269 (64.1%) were information pages.

As shown in Table 2, discussion pages were a minority in each topic, with Vaccinations being the topic less discussed (discussion pages being 10.5% of the total for this topic). On the other hand, Folic Acid and Alcohol were the topics with the highest proportion of discussion pages (48.3% and 43.3%).

**Table 2:** Web pages publication.

**Topic**	**Web pages** ^a^	**Discussion pages** ^b^	**Information pages** ^c^	**ACOG consistent publication pages** ^d^
**Nutrition**	225 (53.6%)	81 (36%)	144 (64%)	58 (40.3%)
**Infectious Diseases**	179 (42.6%)	69 (38.5%)	110 (61.5%)	49 (44.5%)
**Chronic Diseases**	143 (34%)	40 (28%)	103 (72%)	53 (51.5%)
**Genetic Risk**	130 (31%)	39 (30%)	91 (70%)	43 (47.3%)
**Medications**	118 (28.1%)	36 (30.5%)	82 (69.5%)	25 (30.5%)
**Folic Acid**	89 (21.2%)	43 (48.3%)	46 (51.7%)	21 (45.7%)
**Environmental Exposure**	80 (19%)	27 (33.8%)	53 (66.2%)	16 (30.2%)
**Smoking**	66 (15.7%)	17 (25.8%)	49 (74.2%)	18 (36.7%)
**Physical Activity**	61 (14.5%)	12 (19.7%)	49 (80.3%)	22 (44.9%)
**Alcohol**	30 (7.14%)	13 (43.3%)	17 (56.7%)	4 (23.5%)
**Sexually Transmitted Diseases**	29 (6.9%)	8 (27.6%)	21 (72.4%)	6 (28.6%)
**Vaccinations**	19 (4.52%)	2 (10.5%)	17 (89.5%)	11 (64.7%)
**Illicit Drugs**	15 (3.57%)	3 (20%)	12 (80%)	3 (25%)
**Total**	420 (100%)	151 (35.9%)	269 (64.0%)	115 (42.8%)

^a^ Percentage on total number of pages.

^b^ Percentage on topic pages.

^c^ Percentage on topic pages.

^d^ Percentage on topic information pages.

Regarding information pages, Nutrition was the topic with the highest volume of pages, followed by Infectious Diseases and Chronic Diseases. Illicit Drugs, STDs and Vaccinations were those with the lowest web page volume.

Less than 50% of the analyzed information pages reported a complete and correct recommendation, with the lowest figure (23%) for web pages including recommendations on alcohol consumption.

### Sharing on social networks

The 420 web pages containing preconception care contents generated a total of 18,262 interactions on social networks. Facebook was largely the most used social network, with 16,077 (88.0%) interactions, followed by LinkedIn (1,408, 7.71%), Twitter (590, 3.23%), Google+ (181, 0.99%) and other social networks (6, 0.03%).

Table 3 shows the sharing volumes and the sharing/page ratio for each topic.

**Table 3:** Sharing volumes and the sharing per page (s/p) ratio for each topic.

**Topic**	**Total sharing** ^a^	**Sharing per Page ratio**
**Nutrition**	12156 (66.6%)	84.4 s/p
**Infectious Diseases**	9009 (49.3%)	81.9 s/p
**Chronic Diseases**	6385 (35.0%)	62 s/p
**Medications**	5668 (31.0%)	69.1 s/p
**Genetic Risk**	4524 (24.8%)	49.7 s/p
**Folic Acid**	2609 (14.3%)	56.7 s/p
**Vaccinations**	2603 (14.3%)	153 s/p
**Environmental Exposure**	2505 (13.7%)	47.3 s/p
**Physical Activity**	1601 (8.77%)	32.7 s/p
**Smoking**	772 (4.23%)	15.8 s/p
**Illicit Drugs**	319 (1.75%)	26.6 s/p
**Alcohol**	267 (1.46%)	15.7 s/p
**Sexually Transmitted Diseases**	188 (1.03%)	8.95 s/p
**Total**	18262 (100%)	67 s/p

^a^ percentage on total sharing events

Infectious Disease, Chronic Diseases and Nutrition were the most shared topics, while Illicit Drugs, Alcohol and Sexually Transmitted Diseases generated the smallest number of social interactions. Vaccination had the highest sharings/page ratio.

### From monitoring to informing interventions

**Fig 1:**
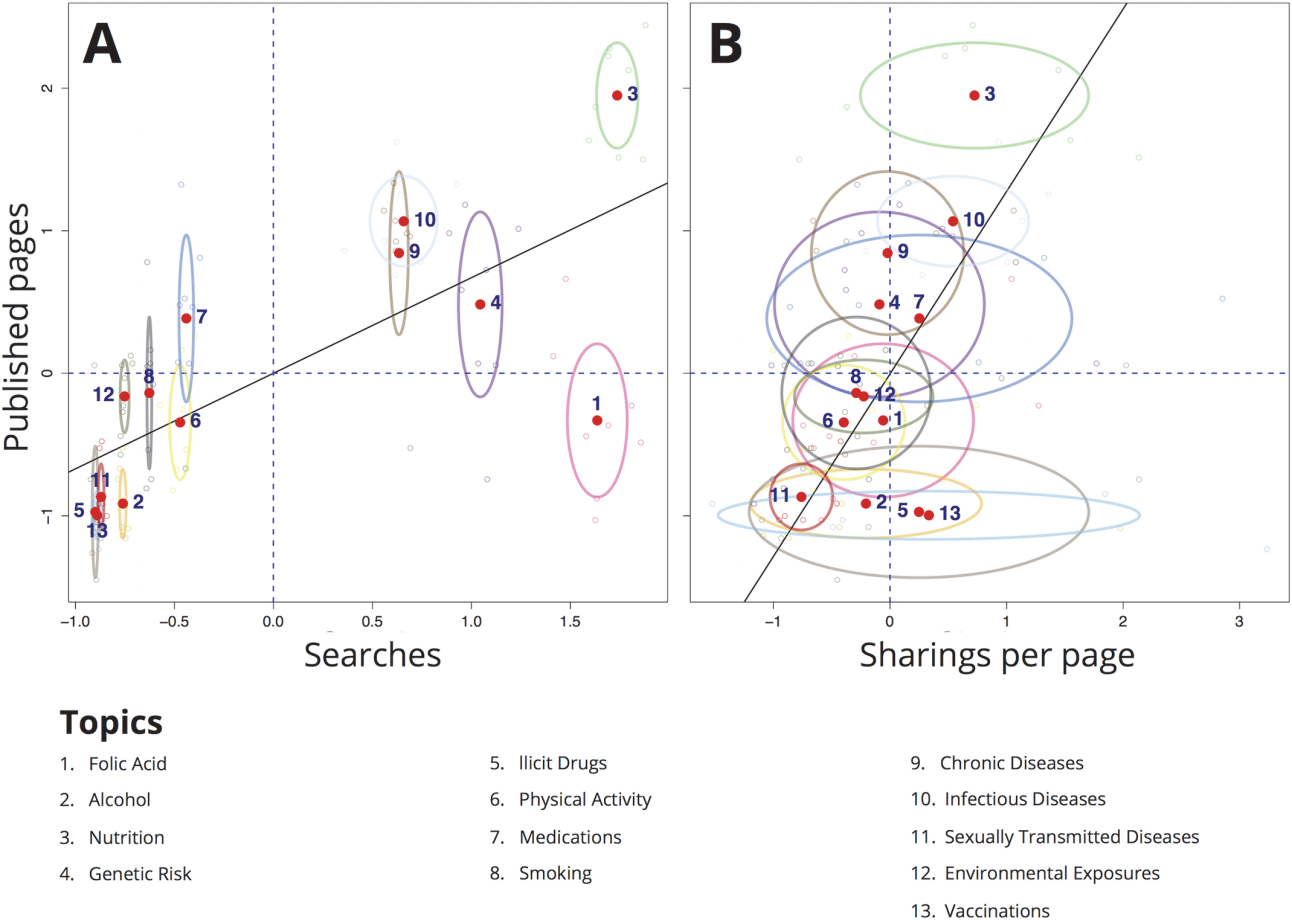
Correlation between the volumes of information pages with searches (A), and with the sharing/page ratio by topic (B).

Fig 1 shows the correlation between the volumes of information pages with Google search volumes (Fig 1A), and with the sharings/page ratio by topic (Fig 1B).

For most topics, the web page volume was correlated with the volume of searches on the same topic (Fig 1A). For Genetic Risk and Folic Acid, a high search volume did not correspond to a high number of web pages, while Medication pages were more frequently published than searched. This analysis allowed to detect topics whose information needs by the public was likely unmet. Overall, topics seemed to be clustered into two separate groups based on search volumes, with a visible gap of interest in between. Variability of correlation over time was modest.

Fig 1B visually describes the relation between the publication volumes and the social network sharing volumes. Nutrition and Infectious Diseases had high publication volumes and high sharings/page ratios, while STDs and Physical Activity had both low publication volumes and sharings/page ratios. Vaccination and Illicit Drugs had high sharings/page ratios despite low volumes of published pages. For some topics, variability of sharing over time was high (as represented by ellipse size). The variability of social network traffic is striking for Vaccinations, a topic associated with a very low and constant number of pages produced.

## Discussion

Our study describes a multi-level, semi-automatic, cost-affordable and dynamic approach to plan data-driven communication strategies for preconception health. We assessed information demand and supply to detect topics that are insufficiently represented on web pages. We also assessed correctness of information on preconception health published on the Internet. Our results represent a resource to prioritize communication on specific topics on the web, to address misconceptions, and to tailor information to specific populations.

Our study shows a reduced interest about the importance of appropriate health behaviors and interventions to be taken before the onset of pregnancy in order to minimize the risk of APOs. As a matter of fact, only few Google searches contained a term which was univocally associated with the preconception period. It is plausible that people search for information on the preconception period including the term “pregnancy” in search queries. Since we did not interview Internet users, we cannot say whether some searches which included the term "pregnancy" were submitted to actually gather information on preconception care. On the other hand, many of the behaviors and interventions recommended during the preconception period are also valid during pregnancy. Preconception health, however, still needs to be promoted as the public tends to take into account preventive actions during pregnancy only.

Many searches and a large volume of published and shared information regarded nutrition and diet. On the other hand, illicit drugs and vaccinations were less frequently searched and published. Nevertheless, web pages containing recommendations on vaccinations were often shared.

We found that less than 50% of web pages regarding nutrition reported information which were consistent with the ACOG guidelines. This finding calls for more efficacious communication programs set by public health agencies and by scientific and academic bodies.

The divergence between search and publication volumes by topic and sharings per page underlined subjects that deserve attention in communication programs, such as folic acid supplementation, genetic diseases and vaccinations.

Information on folic acid still represent a priority. This topic is frequently searched, although correct information is less frequently available on the web. Our results also indicate that the public interest is frequently oriented towards the effect of folic acid during pregnancy rather than in the preconception period.

Chronic diseases, in particular diabetes, hypertension and thyroid diseases also deserve a larger amount of reliable information on the web. Few searches were related to the advantages of performing a genetic risk assessment prior to conception [21, 22]. Safety of medications, in particular of paracetamol and antibiotics, should be carefully considered in a communication program. A topic of interest which is not enough scientifically addressed is the safety of products for personal care (cosmetics, body creams, etc…) during pregnancy. Patients should be warned about the risks of alternative, herbal or imported products which do not meet safety requirements or may contain lead [23]. Among lifestyles, it is worth noting that a large number of users seek for information on the “allowed” number of cigarettes or alcohol drinks during pregnancy, showing that the recommendation of completely quitting smoking and alcohol intake has not been sufficiently acknowledged. Moreover, a notable amount of search queries by Internet users regarded the use of marijuana during pregnancy.

For all topics, our study generated a detailed list of specific subjects that should be addressed and that can be easily translated into communication actions.

Google searches have been previously studied to describe search attitudes of the public regarding health related topics, including breast screening [24], epilepsy [25], bariatric surgery [26], mental health [27], drug utilization [28], safe infant sleep [29] and obesity [30]. On the other hand, presence of reliable health-related information on the web has been previously investigated in different areas, such as influenza [31], vaccinations [32], dental care [33], type 2 diabetes [34], ophthalmic conditions [35] and preconception health [36].

One of the novelties of our system is the approach we used to build our keyword set. Most of the previous studies investigated search behaviors using keywords arbitrarily chosen by the researchers. Instead, we selected our keyword set through an iterative system, based on the Google Adwords platform, which allowed us to identify keywords and queries actually used by Internet users.

Another advantage of the present approach to information need monitoring is the possibility of detecting variations over time of the balance between information demand and supply. This feature offers the possibility to continuously adjust and tailor information interventions according to detected trends and, most importantly, to assess the impact of communication initiatives on specific topics. Finally, a similar approach can be virtually applied to any health topic.

Our approach has a number of limitations.

First, one may argue that Internet use for health contents is not universal. Nevertheless, in Italy, 85,7% of individuals has access to the Internet [37], and it is known that the great majority of women in fertile age use the Internet to retrieve medical information [38].

Another limit concerns the fact that our system implies human classification of contents, which can be time consuming. Moreover, regarding web page analysis, only a small number of the pages identified by our filters was retained after manual review. This is due to the fact that the monitoring system was designed to be sensitive rather than specific, therefore the chosen keywords were aimed at gathering a large number of resources, loosely related to pregnancy and preconception. Actually, a simple keyword-based approach is not powerful enough to distinguish complex pieces of medical information from other pregnancy-related content without human intervention. More complex approaches based on machine learning and natural language processing could reduce this human workload.

Another limit is that the system automatically includes only pages classified as popular by the Alexa system, with a higher probability of being accessed. Thus, web pages with a low traffic have not been considered in the monitoring, although they may actually contain reliable information. Nevertheless, such web pages likely have a trivial effect on population opinion.

Finally, our data is automatically obtained from free Internet tools. This solution allowed us to contain costs. Nevertheless, third-party tools may not be always available over time, thus jeopardizing the reliability of the system.

In conclusion, by analyzing data about Internet usage, our method provides a proof of concept that a cost-affordable, time-efficient and country-specific platform for describing information gaps and needs regarding public health themes may be developed for informing public health interventions.

Through the results our method provides, public health promoters may get insights into the population’s information needs. This would permit data-based strategic choices for the implementation of health promotion campaigns, which would directly target the real interests of the population. Moreover, such a system may allow health operators to anticipate patients’ doubts. Tools like the present one could help spot trends of incorrect information flowing through the web, allowing to counteract such trends as soon they arise. Finally, by analyzing the virality potential of information, health promoters could have real data on which to build social health campaigns.

## Supporting Information

S1 Algorithm Summary of the three components of the study. Chart with a summary of the three components of the study and summary of results.
(PDF)

S1 Supplement Keywords’ list. List of keywords used to detect and filter contents related to ACOG’s guidelines, translated from Italian (grammatic variants have been omitted).
(PDF)

S2 Supplement Google searches sub-topics. List of searches made on Google for each ACOG topic, grouped in sub-topics. The number of searches with percentage on the total, and the number of queries for each sub-topics is reported.
(PDF)

S1 Data Google Search Volumes. (XLSX)

S2 Data Web Production and Sharing. (XLSX)
