# Supplementary material for: Web-Based Surveillance of Public Information Needs for Informing Preconception Interventions
Source: PLoS One. 2015 Apr 16;10(4):e0122551. doi: 10.1371/journal.pone.0122551 (PMC4399910; doi:10.1371/journal.pone.0122551)

13 TOPICS

ACOG Guidelines

1. Alcohol
2. Chronic Diseases
3. Environmental Exposures
4. Folic Acid
5. Genetic Risk
6. Illicit Drugs
7. Infectious Diseases
8. Medications
9. Nutrition
10. Physical Activity
11. Sexually Transmitted Diseases
12. Smoking
13. Vaccinations

We selected 13 topics from the ACOG guidelines for preconception care. For each topic, we selected a set of keywords.

Specific Keywords

- drugs
- barbiturate
- ACE inhibitors
- rethinoids
- valproate
- talidomide
- ...

\+

Moreover we selected 4 basic keywords to filter pregnancy and preconception material

Basic Keywords

pregnancy -
preconception -
prenatal -
conception -

The specific keywords were combined with the basic keywords into "probes".

STUDY PROBES

The probes were amplified using Google Adwords to discover more words and concepts related to our topics, as actually used by Internet users in Google searches.

Google AdWords

We submitted the probes to Google AdWords and retrieved Google search data related to the topics.

Total search volume:	1,995,030
Number of search queries:	1,807
Preconception only search volume:	41,490 (2.08%)

Search queries were then manually reviewed and clustered into 255 subjects to help spotting specific issues of interest.

talkwalkeralerts

RSS → DATABASE

The probes were used to retrieve web pages, which were stored in our database and subsequently filtered.

Retrieved web pages:	15,824
Web pages left after automatic exclusion:	5,717

Web pages were then manually reviewed and classified.

Web pages selected after manual review:	420
Discussion pages:	151
Information pages:	269
Webpages consistent with ACOG recommendations	115

SharedCount

The information pages we found were analyzed with SharedCount to retrieve the number of Social Networks' interactions.

Data was retrieved for the major Social Networks: Facebook, Twitter, Linkedin, Google Plus and others.

Total sharings	18,033
Sharings per Page ratio	67.0 s/p

Facebook:	16,077 (88.0%)
Linkedin:	1,408 (7.71%)
Twitter:	590 (3.23%)
Google Plus:	181 (0.99%)
Other SNs:	6 (0.03%)

Indicators

Topic's data was normalized and then averaged and used to compare the different indicators we studied.

Normalization between topics for each month → Average of month values

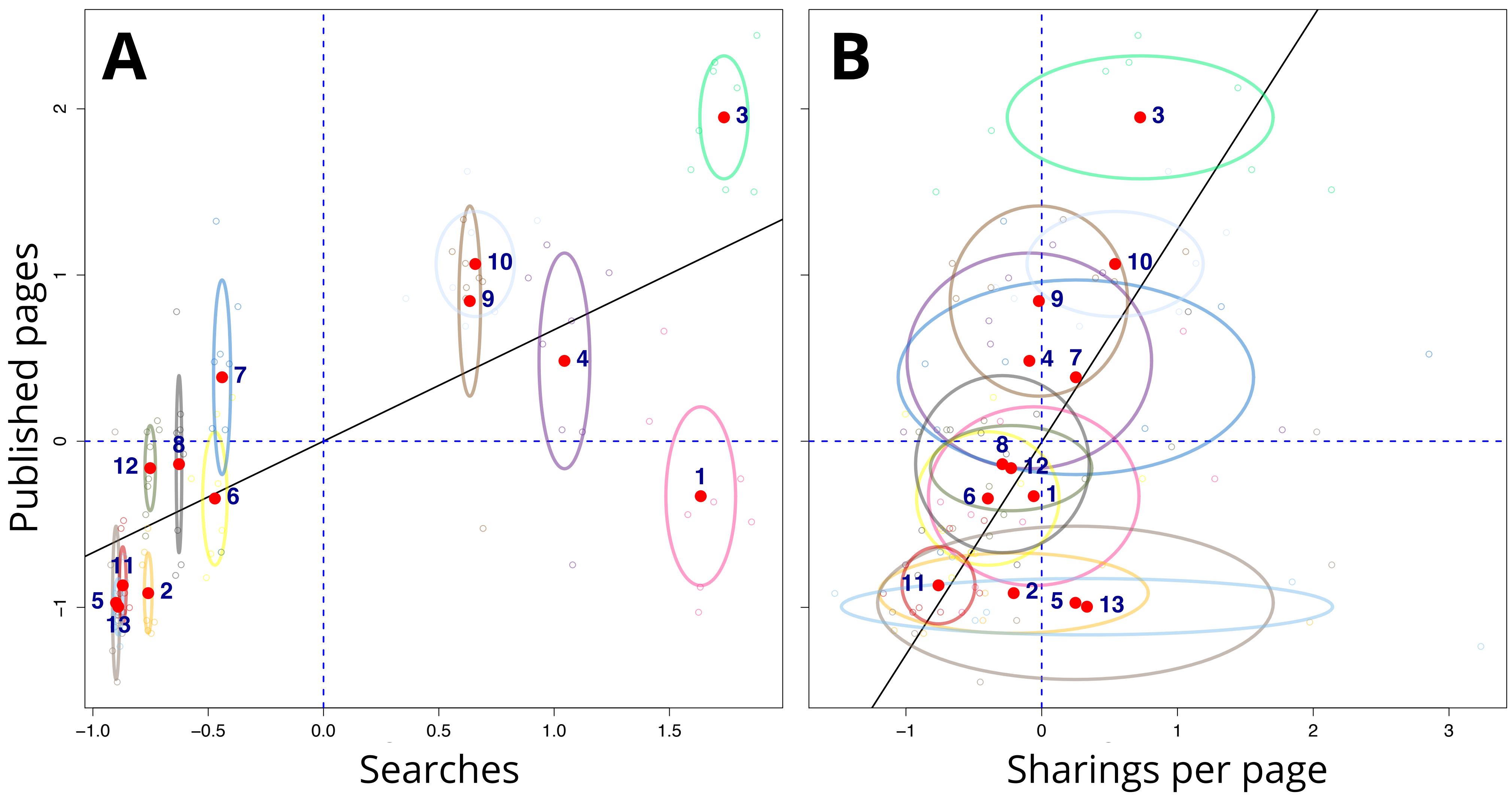

Supplement: S1 Algorithm — Chart with a summary of the three components of the study and summary of results. (PDF) [file pone.0122551.s001.pdf]
