# Supplementary material for: Web-Based Surveillance of Public Information Needs for Informing Preconception Interventions
Source: PLoS One. 2015 Apr 16;10(4):e0122551. doi: 10.1371/journal.pone.0122551 (PMC4399910; doi:10.1371/journal.pone.0122551)
Supplement: S1 Supplement — List of keywords used to detect and filter contents related to ACOG’s guidelines, translated from Italian (grammatic variants have been omitted). (PDF) [file pone.0122551.s002.pdf]

Supplement S1. List of keywords' sets.

List of keywords used to detect and filter contents related to ACOG's guidelines, translated from Italian (grammatic variants have been omitted).

Basic keywords

pregnancy; preconception; conception; prenatal.

Specific keywords related to preconception topics

Alcohol

alcohol; alcoholism; beer; strong drinks; wine.

Chronic Diseases

allergy; anti-phospholipid; arthritis; asthma; asthmatic; cardiovascular; chronic diseases; diabetes; disease; epilepsy; failure; glycemia; hyperphenylalaninemia; hypertension; hyperthyroidism; hypotension; hypothyroidism; infarction; lupus; myocardial; phenylketonuria; phospholipids; pressure; renal; thrombophilia; thyroid; thyroiditis.

Environmental Exposures

cadmium; chemical; chemicals; cosmetics; creams; detergents; ether; ethylene; herbicides; lead; mercury; metals; pesticides; pollution; radiation; radioactive; smog; solvents; toxic; toxic substances; toxicity; toxins; trichloro ethane; wi-fi; wifi; xylene.

Folic Acid

B9; folate; folates; folic; folina.

Genetic Risk

amniocentesis; counseling; diagnosis; diagnostics; genetic counseling; genetic screening; screening; test; villocentesis.

Illicit Drugs

cannabis; cocaine; drug; drug addiction; drugs; hashish; joints (and synonims); marijuana; psychotropics.

Supplement S1. List of keywords' sets.

Infectious Diseases

bordetella; cmv; cytomegalovirus; fifth disease; flu; flu-like symptoms; hand foot mouth disease; hbv; hepatitis; herpes; hsv; infections; infectious; infectious diseases; listeria; listeriosis; pertussis; roseola; rubella; streptococcus; tetanus; toxo; toxoplasma; toxoplasmosis; varicella; virus; VZV; zoster.

Medications

ace inhibitors; analgesic; analgesics; antidepressants; antiepileptic drugs; anti-inflammatory; antibiotic; antibiotics; antidepressant; antidepressants; antiepileptics; antifungal; antitumor; barbiturates; benzodiazepines; carbamazepine; chemotherapy; corticosteroids; drugs; ergot; immunosuppressive; medicaments; methimazole; painkillers; paroxetine; pharmacological; pharmacology; phenobarbital; radiotherapy; retinoids; thalidomide; tiamazolo; valproate; valproic.

Nutrition

aliment; alimentary; almonds; anorexia; chocolate; cholesterol; diet; dukan; eat; fish; food; high protein; hypercaloric; hypocaloric; hypoglicemic; low protein; mushrooms; nutrition; obese; obesity; overweight; salty; shellfish; sweet; sweets; under weight; vegan; veganism; vegetarian; vegetarianism.

Physical Activity

exercise; physical activity; physical exercise; sport.

Sexually Transmitted Diseases

bacteriuria; chlamydia; gonorrhea; hiv; nesseria; sexual disease; sexual infection; sexually transmitted diseases; STDs; syphilis; treponema; vaginitis; vaginosis; venereal.

Smoking

cigarettes; smoke; smoking.

Vaccinations

flu vaccination; flu vaccine; hepatitis b vaccine; hepatitis vaccine; rubella vaccine; tetanus vaccine; vaccinate; vaccinated; vaccination; vaccines.
