# Supplementary material for: Web-Based Surveillance of Public Information Needs for Informing Preconception Interventions
Source: PLoS One. 2015 Apr 16;10(4):e0122551. doi: 10.1371/journal.pone.0122551 (PMC4399910; doi:10.1371/journal.pone.0122551)
Supplement: S2 Supplement — List of searches made on Google for each ACOG topic, grouped in sub-topics. The number of searches with percentage on the total, and the number of queries for each sub-topics is reported. (PDF) [file pone.0122551.s003.pdf]

Supplement S2. Google searches sub-topics.

List of searches made on Google for each ACOG topic, grouped in sub-topics.
The number of searches with percentage on the total, and the number of queries for each sub-topics is reported.

Nutrition

Sub–topics	Searches	Queries
nutrition in general	198460 (44.63%)	189
foods to be avoided	47040 (10.58%)	49
mushrooms	22720 (5.11%)	11
weight control	18530 (4.17%)	19
diabetes and blood sugar control	17350 (3.90%)	18
strawberries	15100 (3.40%)	2
fish	12400 (2.79%)	20
shellfish	10690 (2.40%)	15
food and conception	10490 (2.36%)	42
cocoa and chocolate	10190 (2.29%)	10
milk and dairy products	8860 (1.99%)	6
food source of iron	6740 (1.52%)	8
dietary supplements and conception	4930 (1.11%)	8
desserts and ice cream	4790 (1.08%)	8
dietary supplements	3970 (0.89%)	12
food source of folic acid	3340 (0.75%)	1
lose weight in pregnancy	3090 (0.69%)	12
spicy food	2970 (0.67%)	3
salami and cold meet	2940 (0.66%)	8
coffee	2930 (0.66%)	2
raw fish and sushi	2700 (0.61%)	3
almonds	2460 (0.55%)	3
vegetarian diet	2400 (0.54%)	5
cholesterol	2250 (0.51%)	2
dukan diet	1930 (0.43%)	5
chinese food	1580 (0.36%)	2
obesity	1540 (0.35%)	7
nutrition before pregnancy	1520 (0.34%)	6
toxoplasmosis and food	1520 (0.34%)	7
hypoglycemia	1420 (0.32%)	2
meat	1390 (0.31%)	4
water	1280 (0.29%)	3
vegan diet	1270 (0.29%)	5
fruit servings	1130 (0.25%)	5
mayonnaise	1120 (0.25%)	2
poor diet during pregnancy	1070 (0.24%)	2
salty food	1030 (0.23%)	3

Supplement S2. Google searches sub-topics.

Sub-topics	Searches	Queries
anorexia	1020 (0.23%)	3
diets during pregnancy	1000 (0.22%)	4
calcium intake during pregnancy	940 (0.21%)	2
olives and olive oil	820 (0.18%)	2
high-protein diet	730 (0.16%)	3
maca and conception	630 (0.14%)	2
vegetable servings	620 (0.14%)	3
food poisoning	540 (0.12%)	1
dietician	420 (0.09%)	3
high transaminase levels in pregnancy	300 (0.07%)	1
spoiled food	280 (0.06%)	2
zone diet	260 (0.06%)	1
eggs	260 (0.06%)	1
foods with laxative effects	250 (0.06%)	2
decaffeinated coffee	250 (0.06%)	1
legumes	230 (0.05%)	2
foods for anemia	220 (0.05%)	1
diet for preeclampsia	210 (0.05%)	1
licorice	190 (0.04%)	1
fried food	100 (0.02%)	1

Sub-topics	Searches	Queries
raw food	90 (0.02%)	1
dietary supplements before pregnancy	90 (0.02%)	1
coffee and conception	50 (0.01%)	1
**Total**	**444640**	**549**

Folic Acid

Sub-topics	Searches	Queries
folic acid in general	296610 (69.8%)	18
folic acid during pregnancy	36900 (8.68%)	11
foods that contain folic acid	18490 (4.35%)	18
folic acid drugs	15260 (3.59%)	34
folic acid intake and usage	15050 (3.54%)	40
folic acid properties and functions	13170 (3.10%)	7
folic acid price	8840 (2.08%)	13
folic acid before pregnancy	8810 (2.07%)	9
folic acid side effects	4120 (0.97%)	4
folic acid to get pregnant	3370 (0.79%)	11
folic acid deficiency	2170 (0.51%)	1

Supplement S2. Google searches sub-topics.

Sub–topics	Searches	Queries
folic acid with other supplements	750 (0.18%)	8
spina bifida	680 (0.16%)	3
folic acid contraindications	660 (0.16%)	1
carrying out a pregnancy without folic acid	180 (0.04%)	1
homeopathic folic acid	170 (0.04%)	1
folic acid symptoms	90 (0.02%)	1
**Total**	**425230**	**180**

Genetic Risk

Sub–topics	Searches	Queries
amniocentesis	209480 (63.4%)	49
dual test	22190 (6.72%)	8
chorionic villus sampling	14440 (4.37%)	16
amniocentesis and chorionic villus sampling	10220 (3.09%)	5
examinations for artificial insemination	9710 (2.94%)	10
prenatal diagnosis	9370 (2.84%)	8
examinations for pregnancy	8140 (2.46%)	3

Sub–topics	Searches	Queries
combined screening test (for Down's syndrome)	6930 (2.10%)	7
genetic counseling	5900 (1.79%)	3
screening in pregnancy	5030 (1.52%)	8
examinations pre pregnancy	4310 (1.30%)	9
triple test (for Down's syndrome)	3820 (1.16%)	3
fetal DNA	3270 (0.99%)	3
ultrasonography in pregnancy	3220 (0.97%)	5
diagnostic centers	3150 (0.95%)	9
prenatal cystic fibrosis diagnosis	3110 (0.94%)	3
genetic counseling for pregnancy	2510 (0.76%)	10
nuchal translucency	2270 (0.69%)	7
diagnosis for spina bifida	580 (0.18%)	1
examinations during pregnancy	550 (0.17%)	2
amniocentesis and dual test (for Down's syndrome)	530 (0.16%)	2
diagnosis of Down's syndrome	340 (0.10%)	3
fetal malformations	280 (0.08%)	2

Supplement S2. Google searches sub-topics.

Sub-topics	Searches	Queries
dual and triple test (for Down's syndrome)	210 (0.06%)	1
preconception genetic counseling	200 (0.06%)	1
sca test	170 (0.05%)	1
afp test	170 (0.05%)	1
diagnosis of fragile x	150 (0.05%)	1
amniocentesis and nuchal translucency	110 (0.03%)	1
diagnosis of dwarfism	70 (0.02%)	1
**Total**	**330430**	**183**

Infectious Diseases

Sub-topics	Searches	Queries
toxoplasmosis	44200 (17.2%)	27
cytomegalovirus	41170 (16.0%)	26
cystitis	28140 (10.9%)	24
varicella zoster	25110 (9.76%)	13
streptococcus	24000 (9.33%)	23
herpes viridae	22910 (8.90%)	20
flu	19380 (7.53%)	14

Sub-topics	Searches	Queries
rubella	12390 (4.82%)	5
diarrhea and other bowel disorders	10480 (4.07%)	11
infectious diseases	5180 (2.01%)	14
lower urinary tract infections	4960 (1.93%)	6
listeria	4390 (1.71%)	4
toxoplasmosis and food	3030 (1.18%)	12
vaginal infections	2430 (0.94%)	3
HBV	2260 (0.88%)	4
HCV	1190 (0.46%)	3
pneumonia	1120 (0.44%)	2
fever and conception	1100 (0.43%)	6
hepatitis	790 (0.31%)	2
HAV	540 (0.21%)	1
hemorrhagic cystitis	420 (0.16%)	1
cervical ectropion and conception	420 (0.16%)	1
toxoplasmosis and pets	380 (0.15%)	2
pertussis	360 (0.14%)	1
CMV before pregnancy	250 (0.10%)	1
flu and conception	190 (0.07%)	1

Supplement S2. Google searches sub-topics.

Sub-topics	Searches	Queries
HEV	180 (0.07%)	1
cystitis and conception	140 (0.05%)	1
mumps	110 (0.04%)	1
fever	60 (0.02%)	1
**Total**	**257280**	**231**

Chronic Diseases

Sub-topics	Searches	Queries
gestational Diabetes	67230 (27.8%)	28
glycemia	44790 (18.5%)	38
hypertension	29200 (12.1%)	25
diabetes and blood sugar	16420 (6.79%)	15
diabetes	14780 (6.11%)	21
thyroid	14360 (5.94%)	12
optimum blood pressure	11610 (4.80%)	10
hypothyroidism	10400 (4.30%)	9
hyperthyroidism	6400 (2.65%)	9
thyroiditis	4600 (1.90%)	9
thrombophilia	4600 (1.90%)	6
low blood sugar	2760 (1.14%)	2
asthma	2370 (0.98%)	5
thyroid and conception	1790 (0.74%)	4
allergy	1740 (0.72%)	5
hypothyroidism and conception	1360 (0.56%)	5
epilepsy	1260 (0.52%)	3
chronic diseases in general	1250 (0.52%)	3
arthritis	1230 (0.51%)	4
lupus	990 (0.41%)	2
normal pressure	870 (0.36%)	1
autoimmune diseases	560 (0.23%)	2
hyperthyroidism and conception	400 (0.17%)	3
eclampsia	270 (0.11%)	1
infarction	220 (0.09%)	1
diet for preeclampsia	210 (0.09%)	1
thyroiditis and conception	130 (0.05%)	1
**Total**	**241800**	**225**

Supplement S2. Google searches sub-topics.

Medications

Sub-topics	Searches	Queries
paracetamol	29900 (36.1%)	6
antibiotics	18600 (22.4%)	18
drugs	17100 (20.7%)	39
laxatives	3300 (3.99%)	1
painkillers	2770 (3.35%)	6
paroxetine	1550 (1.87%)	3
anti-inflammatory	1510 (1.82%)	2
magnesium	940 (1.14%)	4
antibiotics and conception	910 (1.10%)	4
antidepressants	870 (1.05%)	3
x-rays	780 (0.94%)	2
radiotherapy	780 (0.94%)	2
chemotherapy	740 (0.89%)	3
cough medications	550 (0.66%)	1
sereupin	430 (0.52%)	1
benzodiazepines	400 (0.48%)	2
homeopathy	370 (0.45%)	2
chemotherapy before pregnancy	290 (0.35%)	1
antinausea	210 (0.25%)	1
antiepileptics	180 (0.22%)	1
corticosteroids	140 (0.17%)	1
antihypertensives	120 (0.15%)	1
radiation therapy before pregnancy	120 (0.15%)	1
thalidomide	110 (0.13%)	1
hormonal therapies	100 (0.12%)	1
**Total**	**82740**	**107**

Physical Activity

Sub-topics	Searches	Queries
effects or whether it is safe to play sport	23630 (30.3%)	31
specific exercises for pregnancy	22280 (28.6%)	17
yoga	15500 (19.9%)	15
water gymnastics	8330 (10.7%)	5
pilates	7460 (9.58%)	4
sport and conception	380 (0.49%)	2
yoga and conception	300 (0.39%)	2
**Total**	**77880**	**76**

Supplement S2. Google searches sub-topics.

Smoking

Sub-topics	Searches	Queries
smoking in pregnancy	35320 (68.4%)	19
how many cigarettes can be smoked	4380 (8.48%)	7
electronic cigarettes	3730 (7.22%)	7
effects of smoking in pregnancy	3370 (6.52%)	11
quit smoking for pregnancy	3110 (6.02%)	5
passive smoking	1060 (2.05%)	1
smoke and conception	690 (1.34%)	3
**Total**	**51660**	**53**

Alcohol

Sub-topics	Searches	Queries
alcohol in general	9530 (32.5%)	16
wine	8990 (30.6%)	10
beer	8170 (27.8%)	10
non-alcoholic beer	1100 (3.75%)	1
how much wine in pregnancy	810 (2.76%)	2
alcohol risks for pregancy	270 (0.92%)	2
fetal alcohol syndrome	220 (0.75%)	2
how much beer in pregnancy	170 (0.58%)	2
alcoholism	80 (0.27%)	1
**Total**	**29340**	**46**

Environmental Exposures

Sub-topics	Searches	Queries
cosmetic creams	9050 (31.1%)	26
almond oil	7760 (26.6%)	7
cellulite creams	4040 (13.9%)	7
depilatory creams	2130 (7.31%)	2
cosmetics	1020 (3.50%)	4
hair dye	880 (3.02%)	3
sunscreens	750 (2.57%)	8
cleansers	690 (2.37%)	3
x-rays	570 (1.96%)	4
mobile phone signals	420 (1.44%)	2
body oils	420 (1.44%)	1
wifi signals	400 (1.37%)	1
radiation	330 (1.13%)	1

Supplement S2. Google searches sub-topics.

Sub–topics	Searches	Queries
anti–age creams	280 (0.96%)	2
perfumes	160 (0.55%)	1
toxic substances	140 (0.48%)	1
mercury	90 (0.31%)	1
**Total**	**29130**	**74**

Sexually Transmitted Diseases

Sub–topics	Searches	Queries
vaginitis	2980 (27.6%)	4
syphilis	1830 (16.9%)	4
HIV	1490 (13.8%)	5
chlamydia	1140 (10.6%)	2
cystitis and conception	870 (8.06%)	3
hiv test	730 (6.76%)	3
bacteriuria	680 (6.30%)	3
vaginosis	380 (3.52%)	2
gonorrhea	220 (2.04%)	2
candida	190 (1.76%)	1
gardnerella and conception	120 (1.11%)	1
HPV and conception	90 (0.83%)	1

Sub–topics	Searches	Queries
vaginitis and conception	80 (0.74%)	1
**Total**	**10800**	**32**

Vaccinations

Sub–topics	Searches	Queries
rubella vaccine	2800 (36.3%)	3
vaccinations	1400 (18.1%)	8
rubella vaccine before pregnancy	770 (9.97%)	2
varicella vaccine	700 (9.07%)	2
tetanus vaccine	300 (3.89%)	2
HBV vaccine	230 (2.98%)	2
influenza vaccine	1170 (15.2%)	5
trivalent vaccine	190 (2.46%)	1
malaria prophylaxis	160 (2.07%)	1
**Total**	**7720**	**26**

Illicit Drugs

Sub–topics	Searches	Queries
marijuana	4860 (76.18%)	16

Supplement S2. Google searches sub-topics.

Sub-topics	Searches	Queries
cocaine	800 (12.54%)	4
drugs	400 (6.27%)	3
methadone	210 (3.29%)	1
drug addiction	110 (1.72%)	1
**Total**	**6380**	**25**
